# Ready-to-Eat Cereal Consumption Patterns: The Relationship to Nutrient Intake, Whole Grain Intake, and Body Mass Index in an Older American Population

**DOI:** 10.1155/2012/631310

**Published:** 2012-10-10

**Authors:** Ann M. Albertson, A. Christine Wold, Nandan Joshi

**Affiliations:** ^1^General Mills: Bell Institute of Health and Nutrition, James Ford Bell Technical Center, 9000 Plymouth Avenue North, Minneapolis, MN 55427, USA; ^2^General Mills: Quality & Regulatory Operations, 1 General Mills Boulevand, BT 10-D-1, Minneapolis, MN 55426, USA; ^3^General Mills India Pvt. Ltd., 601-Prudential, Hiranandani Business Park, Powai, Mumbai 400076, India

## Abstract

*Objective*. To investigate the relationship between ready-to-eat (RTE) breakfast cereal consumption patterns and body mass index (BMI), nutrient intake, and whole grain intake in an older American population. *Design*. A cross-sectional survey of US households, collected by the NPD Group via the National Eating Trends (NET) survey. Main outcome measures include BMI, nutrient intake, and whole grain intake. *Subjects/Setting*. The sample included 1759 participants age 55 and older, which was divided into approximate quartiles based on intake of RTE breakfast cereal for the 2-week period (0 servings, 1–3 servings, 4–7 servings, and ≥8 servings). *Results*. In the multivariate linear regression analysis adjusted for energy and age; intake of dietary fiber, whole grains, and the majority of micronutrients examined were found to be positively associated with frequent RTE cereal consumption. The proportion of participants consuming less than the Estimated Average Requirement (EAR) was lower for the highest quartile of RTE cereal consumers compared to nonconsumers, for the majority of vitamins and minerals examined. Significant differences in BMI between RTE breakfast cereal intake groups were found for men. *Conclusion*. Results suggest that ready-to-eat breakfast cereals may contribute to the nutritional quality of the diets of older Americans. Prospective studies and experimental trials are needed to better evaluate the role of RTE cereal consumption in energy balance.

## 1. Introduction

According to US Department of Health and Human Services, Administration on Aging population estimates, the number of older Americans is expected to grow dramatically. By 2030, the number of adults aged 60+ is expected to increase to approximately 25% of the population, while the number of the oldest old (age 85+) is expected to double [1]. Adequate nutrition is an important factor in continued health and independence for older adults [2, 3]. 

Homebound elderly people who skip breakfast are more likely to have inadequate nutrient intakes [2]. Caloric needs typically drop with advancing age, while needs for certain micronutrients such as calcium, vitamin D, and vitamin B_12_ increase [4]. Thus the importance of choosing nutrient dense foods increases for this population. Several cross-sectional studies have found positive associations between breakfast consumption and RTE breakfast cereal consumption, and increased nutrient intake for adult and elderly adult populations [5–8]. RTE cereal provides a convenient and easy to prepare breakfast option, which can provide whole grains and dietary fiber, along with many vitamins and minerals. RTE breakfast cereal is also an important contributor of whole grain in the US diet, providing approximately 30% of daily whole grain intake [9]. Recently, whole grain intake was found to be inversely associated with BMI and abdominal body fat in an older American population [10]. 

Similar to other age groups, the prevalence of overweight and obesity in the elderly population is increasing [11]. An estimated 76.5% of men and 73.5% of women ≥60 years old are currently overweight or obese (BMI ≥ 25) while 36.6% of men and 42.3% of women ≥60 years old are estimated to be obese (BMI ≥ 30) [12]. Based on recent NHANES data, McKeown estimates 62.5% of men and 74.9% of women ages 60 to 69 years old are abdominally obese [10]. It has been suggested that abdominal obesity may be a better measure of obesity than BMI in an older population, since after the age of 60 this population tends to lose lean body mass, while increasing visceral body fat [13]. 

A number of cross-sectional studies have examined the association of ready-to-eat (RTE) cereal intake with nutrient intake and body weight status. RTE cereal consumption has been found to be positively associated with intake of a range of vitamins and minerals and inversely associated with body mass index (BMI) in child and adolescent populations [14, 15]. In addition, eating breakfast along with a pattern of frequent ready-to-eat cereal consumption is associated with lower BMI, higher nutrient intakes, and weight maintenance in adults [7, 16–19]. Research in older adults is lacking. Thus, we examined the association of RTE cereal consumption patterns with nutrient intake in a sample of older adults who participated in a cross-sectional survey. The association of RTE cereal consumption with BMI and percent overweight or obese (BMI ≥ 25) was also examined in this study.

## 2. Methods

Data collected as part of the National Eating Trends (NET) survey were analyzed. The NET survey is conducted by the NPD Group, a marketing information company that has been monitoring the intake of US households since 1980 through this survey [20]. As described previously, the General Mills Bell Institute of Health and Nutrition (BIHN) has developed a proprietary method for combining data from the NET survey's 14-day food diary data, with portion size estimates from the National Health and Nutrition Examination Survey (NHANES) 1999–2004, and nutrient and food group data from the University of Minnesota's Nutrient Data System for Research (NDSR) Version 34, 2008 (Nutrition Coordinating Center, Minneapolis, MN) [14]. This combined dataset allows for population based, food pattern comparisons of usual intake, based on categories of foods consumed.

### 2.1. Food Consumption Data

Annually, the NET survey samples 2,000 households representing approximately 10,000 individuals, and data from the March 2006 through February 2008 survey were used for this study. To be included in this study, the subjects must have completed at least 7 days of food reporting and have provided height and weight data for calculation of BMI. The NET survey included 1905 participants age 55 and older, of these 1759 completed at least 7 days of food diaries and provided height and weight data (92%). Of these 1759 participants, 46% were male, 54% were female, and 22% were ≥75 years old.

For the NET survey, each year the NPD Group collaborates with a third party to establish a mail access panel that is a cross-sectional group of US households (noninstitutionalized people in the contiguous 48 states). Survey targets are set for the following demographic factors (family versus nonfamily, age, household income, household size, age of head of household, employment status, race, and census region) based on US Census Bureau statistics. NPD uses historical response data to over-sample households with lower expected response rates and adjusts the target levels quarterly to obtain a demographically and geographically balanced sample. Panelists are recruited using mailing lists and mall interventions, and NET survey panelists are then randomly selected from active mail panelists (those who have returned at least 1 other survey). Participant incentives include selection from a range of gifts ($25 to $30 value). Approximately 72% of households provide completed surveys for at least 10 of the 14 days of the annual survey.

Recruitment and data collection were conducted across all 52 weeks of the year. Each household member was required to document food and beverage intake by maintaining a daily eating diary. The “person most responsible for meal preparation” was responsible for documenting the name and brand of all food consumed for all household members, including any additions with cooking or food preparation. Description of the meal occasion (breakfast, lunch, snack, etc.) and location (at home, away from home) was included in the diary along with food names, flavor descriptors, brand names, package types, product form, special nutritional attributes, and other details. Food diary records were mailed back on a daily basis for the 14 day period. 

### 2.2. Food Diary Data Entry

The University of Minnesota's Nutrition Database System for Research (NDS-R) provided the nutrient and food group data for this study. The NDS-R system provides a comprehensive database of complete nutrient data for 156 nutrients or components, for over 18,000 foods. The database is updated on an ongoing basis and contains over 7,000 brand name products. NDS-R has over 100 food groups in the database and each food is assigned a food group. Completed food diaries were entered into NDS-R for calculation of nutrient intake and food groups. Foods reported by NET survey panelists were matched, based on food diary descriptions to foods included in the NDS-R database. The recipe module of the NDS software was used to assign nutrient values to foods with special attributes (low fat, calcium fortified, reduced sodium, etc.) which were not found in the database and each recipe was assigned an appropriate food group. 

### 2.3. Portion-Size Data

NET panelists record the foods and beverages consumed by household members but not the quantities. This procedure is standard for panel surveys to minimize recorder burden and thus increase reliability. Portion-sizes were estimated by combining data from the NHANES 1999–2004. Serving weights for individual food codes were aggregated and then collapsed for like-foods to strengthen cell sizes, and smoothed to eliminate outliers. Age and gender-specific mean serving weights were thereby determined for over 800 food types; these portions were subsequently assigned to each food recorded and coded in the NET diary.

### 2.4. Data Tabulation

Of the 1905 older American adults who were included in the NET survey, 146 participants were excluded for not meeting the inclusion criteria. 94% of those in the analytic sample provided complete (14 days) food diaries. For the 6% of participants with 7 to 13 days of dietary information the estimated servings of RTE cereal were normalized to 14 to allow for placement in the study quartiles. The pattern established over the 7- to 13-day period was assumed to be constant for the full 14 days. For example if no RTE cereal was consumed in the days collected, no servings would be included for the full 14 days, or if 2 servings were consumed in 7 days, 4 servings would be assumed for the full 14-day period. Self-reported values for height and weight were provided by each individual in the study. BMI was calculated by using the formula: BMI = weight (lb)/height (in)^2^ × 703.

Servings of whole grains consumed by study participants were estimated based on food group assignments in the NDS-R database. All grain-based foods in the NDSR database are categorized as being either “whole grain,” “some whole grain” or “refined grain”. To be assigned to the “whole grain” category the first grain ingredient in the product must be a whole grain. Foods assigned to the “some whole grain” category are products that contain whole grain ingredients, but the first grain ingredient is not a whole grain. “Refined grain” products are those that contain no whole grain ingredients. In this paper servings of whole grains were calculated by summing serving of foods classified as “whole grain” in the database.

### 2.5. Statistical Analysis

To examine whether RTE cereal consumption was associated with BMI, body weight status, whole grain, and nutrient intake a series of analysis of variance (ANOVA) models were conducted by approximate quartiles of RTE cereal intake. Slightly greater than one quarter of the participants were nonconsumers, which made breaks in intake levels approximate rather than exact quartiles of RTE cereal intake (Table 1).

Logistic regression modeling was conducted to examine the association between RTE cereal consumption pattern and odds of falling below Estimated Average Requirement (EAR) for micronutrients with an established intake level. Covariates included within the ANOVA and logistic regression models were energy and age. Values were adjusted for energy to see if RTE cereal consumption was associated with a more nutrient dense diet. Age was included as a covariate, because it is associated with both RTE cereal consumption and nutrient intake. The contrasts were examined between the possible pairs of cereal consumption categories using the Wald chi-square test.

Logistic regression was used to compare odds of being overweight or obese by level of RTE cereal consumption. The contrasts were examined between the possible pairs of cereal consumption categories using the Wald chi-square test. For both the ANOVA and logistic regression models examining the association of RTE cereal consumption with body weight age and age^2^ were included as covariates. Age^2^ was included because the relationship between BMI and age is not linear, around 60 years of age this relationship can change [13]. All analyses were stratified by sex, because it was hypothesized that the association of RTE cereal consumption with the measured outcomes may vary by sex.

An alpha level of 0.01 was used to determine the significance for the analysis of variance comparisons, except where otherwise noted. All analyses were performed using SAS version 9.2 (SAS Institute, Cary, NC).

## 3. Results

The sample size and RTE cereal consumption distribution are displayed in Table 1. With respect to findings related to food and nutrient intake, the objective of this study was to determine the relationship between RTE cereal quartile of consumption to nutrient intake. For both men and women in the sample, significant differences in nutrient intakes between the fourth quartile (Q4) compared to the lowest two quartiles (Q1 and Q2) of RTE cereal consumption were seen for most nutrients examined (Table 2). For males, Q4 intake of energy was higher compared to Q1 and Q2. After adjusting for energy and age there was a significantly lower (*P* < 0.01) intake of total fat, total saturated fat, total trans fat, and cholesterol among those in Q4 compared to Q1 and Q2. In addition among men, intakes of total carbohydrate, dietary fiber, total sugar, vitamin A, vitamin C, vitamin D, thiamin, riboflavin, niacin, vitamin B6, folate, vitamin B12, potassium, calcium, iron, magnesium, and zinc were significantly higher (*P* < 0.01) among those in Q4 compared to those in Q1 and Q2. There were no significant differences between intake of total protein, added sugar, or vitamin E across consumption patterns of RTE cereal. Sodium intake was not significantly different between the highest and lowest quartiles of intake. Similar relationships were found for women age 55+ (Table 3), with the exception of total sugar intake which was not significantly different across quartiles of RTE cereal intake.

In addition to significant differences in nutrient intake, this study found whole grain intake to be positively associated with RTE cereal consumption. For both men and women, average whole grain intake among those in Q4 of RTE cereal consumption was nearly double the whole grain intake of those in the Q1 (the nonconsumers) (Tables 2 and 3).

The percentage of participants in each quartile that fell below Estimated Average Requirement (EAR) for nutrients with established EAR amounts was examined (Table 4). A significant inverse linear relationship was found between the frequency of RTE cereal consumption and the percentage of participants failing to consume the EAR for a number of nutrients. In women, this relationship was found for all nutrients examined except vitamin D and E and for men, all but niacin and iron.

In examining the relationship of RTE cereal consumption patterns and BMI there were some significant differences found in mean BMI for all men and all women (Table 5). With respect to men, the adjusted mean BMI was significantly lower among those in Q4 compared to Q1 and Q2 (*P* = 0.0332). For women, there were significant differences between Q3 and Q4, but no significant differences between the highest and lowest quartiles of RTE cereal intake. 

When comparing the rates of overweight and obesity across RTE cereal consumption levels in analysis adjusted for age and age^2^, significant differences in mean % overweight or obese were found for males but not for females (Table 5). Among men, the rate of overweight and obesity (BMI ≥ 25) was lower among those in Q4 compared to Q1 and Q2 (*P* = 0.0433). Among men, the rate of obesity (BMI ≥ 30) was lower among Q4 compared to Q2. However, no significant differences were observed in other groups. For women, there were no significant differences between quartiles. Results were similar in analysis adjusted for age only (data not shown). 

## 4. Discussion

Study results suggest that frequent RTE cereal consumption may be associated with better nutrient intake profiles and higher whole grain intake in older Americans. Among older men RTE cereal consumption may be associated with lower BMI. Key nutrients that are typically underconsumed in older American populations include protein, fiber, vitamins B12, D, and E, calcium, magnesium, and zinc. Nutrients for which overconsumption is a concern include fat, cholesterol, vitamin A, iron, and zinc. Frequent consumption of RTE cereal was found to be associated with higher intake of all key underconsumed nutrients expect protein, which was not significantly different between the groups. In addition, consumption of whole grains for Q4 was more than double than the Q1 (nonconsuming group). RTE cereal consumption was also associated with lower energy adjusted fat and cholesterol intakes.

It was not surprising to find the positive association between higher RTE cereal consumption with higher intakes of vitamins and minerals, as many RTE cereals are fortified with vitamins and minerals and other studies have found similar associations for both children and adults [7, 14, 15, 17, 21]. In addition, RTE cereal consumption is associated with greater intake of milk and calcium, as milk and RTE cereal are usually consumed together [19]. 

Breakfast intake and frequent RTE cereal consumption have been associated with lower BMI based on a number of cross-sectional studies [14–17]. However, BMI is not an ideal measure of body fatness or mortality risk for older individuals and several studies suggest abdominal circumference may be a better measure of overweight and obesity in an elderly population [13, 22]. This study has data only on BMI as no measure of waist circumference was gathered. In this study, significant associations between RTE cereal consumption and body weight were found only for men. This finding differs from Song's data for adults' ≥19 years of age, which found an inverse association between RTE cereal consumption and BMI for women but not men [17]. 

There are a number of strengths of this study. First, while many studies have data from only 2 to 4 days of food records; this study is based on 14 days of data. Measurement error due to within person coefficient of variation in food and nutrient intake is reduced in this study due to the collection of 14 days of dietary intake information. This study included a representative sample of an older US population. The sample size allowed for examining the association of RTE cereal consumption with nutrient intake and weight status in older adults, with analysis conducted separately for males and females. In addition, the nutrient and food group database NDSR is one of the most comprehensive databases available for diet research, including nutrients of interest such as vitamin D and few missing nutrient values.

One limitation of this study is that a number of factors that could potentially confound the association of RTE cereal with BMI were not assessed and therefore could not be adjusted for in the analysis. For example, it did not include any measure of physical activity or ability to perform daily activities. Therefore, we do not know if this consumption pattern was associated with other lifestyle patterns, such as higher levels of physical activity. Another limitation is that BMI is based on self-reported measures of height and weight, and this is not the most reliable measure since people tend to overestimate their height and underestimate weight. Another limitation is the lack of reported portion size data. Reporting of portion size is one of the most error prone areas of dietary assessment. Portion size values were imputed from NHANES data where respondents are trained and use a variety of estimation tools however; reporting error is a potential issue. Finally, data on intake of dietary nutrient supplements was not gathered. Consequently, the association of RTE cereal consumption with nutrient intake could not be examined in the context of vitamin and mineral intake for all sources. It is unknown if supplementation may help bridge the gap between usual nutrient intakes and dietary recommendations. RTE cereals are generally fortified and can contribute a significant proportion of nutrients to those who consume them.

Overconsumption of certain vitamins and minerals such as vitamin A, iron, and zinc can be a risk for older Americans who eat fortified products and use vitamin and mineral supplements [23]. A recent study found 37% of men and 47% of women age ≥51 years consume at least one vitamin or mineral supplement each day [23]. Data from the 1999-2000 NHANES found 63% of individuals ≥60 years in age consume some type of daily dietary supplements [24]. Many older Americans may be unaware of the risk of overconsumption of vitamins and minerals and more education about this concern is needed. In addition, food manufacturers need to consider this concern for the elderly population when deciding on how to fortify RTE cereal products.

The food grouping estimates in the NDSR database are not ideal of determining whole grain intake. Food items that were grouped as “some whole grain” were not included in this study due to the uncertainty of the amount of whole grain in the food. Foods grouped as “whole grain” were counted as all whole grain, when the food may have other ingredients and also may include some refined grains. The finding of higher levels of whole grain in the highest quartile of RTE cereal consumption makes sense considering the higher intake of fiber for this group and since RTE cereal is an important contributor of whole grain in the US diet.

## 5. Conclusion

Results suggest that ready-to-eat breakfast cereals may contribute to the nutritional quality of the diets of older Americans. Prospective studies and experimental trials are needed to better evaluate the role of RTE cereal consumption in energy balance.

## Figures and Tables

**Table 1:** Sample size and RTE cereal consumption distribution for adults 55+ from the 2007-2008 NET survey.

Gender/age	Sample size	Quartile of cereal consumption			
		Q1	Q2	Q3	Q4
		0 Servings/14 d	1–3 Servings/14 d	4–7 Servings/14 d	8+ Servings/14 d
		*n* (%)	*n* (%)	*n* (%)	*n* (%)
All adults ages 55+	1759	490 (27.9)	484 (27.5)	380 (21.6)	405 (23.0)
Males ages 55+	803	231 (28.8)	207 (25.8)	165 (20.6)	200 (24.9)
Females ages 55+	956	259 (27.1)	277 (29.0)	215 (22.5)	205 (21.4)

**Table 2:** Adjusted* mean daily nutrient intake for adult men, ages 55+, by quartile of cereal consumption.

	Quartile of RTE cereal consumption				
	Q1	Q2	Q3	Q4	
Nutrient	0 Servings	1–3 Servings	4–7 Servings	8+ Servings	*P*
	Mean ± SE	Mean ± SE	Mean ± SE	Mean ± SE	
Energy (kcal)	1826 ± 38^a^	1932 ± 39^ab^	2033 ± 45^bc^	2142 ± 43^c^	<0.0001
Total fat (g)	81 ± 1.6^a^	80 ± 1.8^a^	76 ± 2.0^b^	73 ± 1.9^c^	<0.0001
Total saturated fat (SFA) (g)	27 ± 0.6^a^	27 ± 0.7^a^	25 ± 0.8^b^	25 ± 0.7^b^	<0.0001
Total trans Fat (TRANS) (g)	5.2 ± 0.1^a^	5.3 ± 0.1^a^	5.0 ± 0.2^a^	4.4 ± 0.2^b^	<0.0001
Cholesterol (mg)	307 ± 8^a^	309 ± 8^a^	272 ± 8^b^	244 ± 6^c^	<0.0001
Total carbohydrate (g)	228 ± 5^a^	233 ± 5^a^	246 ± 7^b^	253 ± 6^b^	<0.0001
Dietary fiber (g)	16 ± 0.4^a^	16 ± 0.4^a^	18 ± 0.5^b^	19 ± 0.5^b^	<0.0001
Total sugar (g)	104 ± 4^a^	104 ± 3^a^	113 ± 4^b^	117 ± 4^b^	<0.0001
Added sugar (g)	69 ± 2^a^	67 ± 2^a^	68 ± 2^a^	67 ± 2^a^	0.7652
Total protein (g)	78 ± 1^a^	78 ± 1^a^	77 ± 2^a^	80 ± 1^a^	0.2402
Vitamin A (mcg rae)	735 ± 33^a^	724 ± 30^a^	805 ± 36^ab^	867 ± 29^b^	0.0020
Vitamin C (mg)	81 ± 3^a^	85 ± 4^a^	94 ± 4^ab^	99 ± 4^b^	0.0011
Vitamin D (mcg)	4.2 ± 0.2^a^	4.7 ± 0.2^ab^	5.3 ± 0.2^b^	6.5 ± 0.2^c^	<0.0001
Vitamin E (mg alpha-tocopherol)	6.5 ± 0.2^a^	6.6 ± 0.2^a^	7.3 ± 0.3^a^	7.3 ± 0.3^a^	0.0245
Thiamin (mg)	1.6 ± 0.03^a^	1.7 ± 0.03^b^	1.8 ± 0.04^c^	1.9 ± 0.04^d^	<0.0001
Riboflavin (mg)	2.1 ± 0.04^a^	2.2 ± 0.04^a^	2.4 ± 0.05^b^	2.6 ± 0.05^c^	<0.0001
Niacin (mg)	21.9 ± 0.4^a^	22.5 ± 0.4^a^	23.5 ± 0.5^b^	25.8 ± 0.5^c^	<0.0001
Vitamin B6 (mg)	1.6 ± 0.03^a^	1.7 ± 0.03^a^	1.9 ± 0.04^b^	2.3 ± 0.06^c^	<0.0001
Folate (mcg)	437 ± 8^a^	491 ± 9^b^	588 ± 12^c^	743 ± 20^d^	<0.0001
Vitamin B12 (mg)	5.1 ± 0.2^a^	5.3 ± 0.2^a^	5.6 ± 0.2^a^	7.0 ± 0.2^b^	<0.0001
Sodium (mg)	3509 ± 59^ab^	3584 ± 67^a^	3450 ± 75^ab^	3428 ± 69^b^	0.0146
Potassium (mg)	2544 ± 52^a^	2599 ± 51^a^	2775 ± 65^b^	2875 ± 61^b^	<0.0001
Calcium (mg)	759 ± 21^a^	781 ± 20^a^	895 ± 28^b^	987 ± 30^c^	<0.0001
Iron (mg)	13 ± 0.3^a^	14 ± 0.3^b^	16 ± 0.3^c^	19 ± 0.4^d^	<0.0001
Magnesium (mg)	248 ± 5^a^	252 ± 5^a^	274 ± 6^b^	294 ± 6^c^	<0.0001
Zinc (mg)	10.2 ± 0.2^a^	10.2 ± 0.2^ab^	10.8 ± 0.3^b^	12.3 ± 0.3^c^	<0.0001
Whole grains (servings/14 d)	5.4 ± 6^ a^	6.8 ± 5^ a^	8.8 ± 6^b^	13.0 ± 8^c^	<0.0001

^a,b,c,d^Means within the same row with the same letter are not significantly different (*P *< 0.01). ^∗^Adjusted for energy and age.

**Table 3:** Adjusted* mean daily nutrient intake for adult women, ages 55+, by quartile of RTE cereal consumption pattern.

	Quartile of RTE cereal consumption				
	Q1	Q2	Q3	Q4	
Nutrient	0 Servings	1–3 Servings	4–7 Servings	8+ Servings	*P*
	Mean ± SD	Mean ± SD	Mean ± SD	Mean ± SD	
Energy (kcal)	1363 ± 24^a^	1510 ± 28^b^	1570 ± 32^b^	1622 ± 33^b^	<0.0001
Total fat (g)	60 ± 1.1^a^	60 ± 1.2^a^	58 ± 1.4^b^	55 ± 1.4^c^	<0.0001
Total saturated fat (SFA) (g)	20.0 ± 0.4^a^	20.0 ± 0.5^a^	19.3 ± 0.5^ab^	18.7 ± 0.5^b^	<0.0001
Total trans fat (TRANS) (g)	4.0 ± 0.1^a^	4.0 ± 0.1^a^	3.7 ± 0.1^b^	3.3 ± 0.1^c^	<0.0001
Cholesterol (mg)	216 ± 6^a^	227 ± 5^a^	212 ± 6^a^	188 ± 5^b^	<0.0001
Total carbohydrate (g)	183 ± 3^a^	185 ± 4^ab^	190 ± 4^bc^	197 ± 5^c^	<0.0001
Dietary fiber (g)	13 ± 0.3^a^	13 ± 0.3^a^	13 ± 0.3^a^	15 ± 0.4^b^	<0.0001
Total sugar (g)	88 ± 2^a^	87 ± 3^a^	91 ± 3^a^	92 ± 3^a^	0.0654
Added sugar (g)	58 ± 1^a^	56 ± 1^a^	56 ± 2^a^	53 ± 2^a^	0.1569
Total protein (g)	58 ± 1^a^	59 ± 1^a^	60 ± 1^a^	61 ± 1^a^	0.0648
Vitamin A (mcg rae)	588 ± 21^a^	578 ± 18^a^	631 ± 22^ab^	679 ± 21^b^	0.0005
Vitamin C (mg)	71 ± 3^a^	68 ± 3^a^	76 ± 3^ab^	84 ± 3^b^	0.0001
Vitamin D (mcg)	3.2 ± 0.1^a^	3.8 ± 0.1^b^	4.2 ± 0.1^b^	4.9 ± 0.1^c^	<0.0001
Vitamin E (mg alpha-tocopherol)	5.5 ± 0.2^a^	5.2 ± 0.2^a^	5.8 ± 0.2^a^	5.9 ± 0.2^a^	0.0336
Thiamin (mg)	1.20 ± 0.02^a^	1.26 ± 0.02^b^	1.34 ± 0.03^c^	1.49 ± 0.03^d^	<0.0001
Riboflavin (mg)	1.5 ± 0.03^a^	1.7 ± 0.03^b^	1.8 ± 0.04^c^	2.0 ± 0.04^d^	<0.0001
Niacin (mg)	16 ± 0.3^a^	17 ± 0.3^a^	18 ± 0.3^b^	20 ± 0.4^c^	<0.0001
Vitamin B6 (mg)	1.2 ± 0.02^a^	1.3 ± 0.02^a^	1.5 ± 0.03^b^	1.8 ± 0.05^c^	<0.0001
Folate (mcg)	344 ± 6^a^	384 ± 7^b^	471 ± 10^c^	600 ± 16^d^	<0.0001
Vitamin B12 (mg)	3.7 ± 0.1^a^	4.0 ± 0.1^ab^	4.4 ± 0.1^b^	5.7 ± 0.2^c^	<0.0001
Sodium (mg)	2640 ± 43^a^	2697 ± 45^a^	2622 ± 50^a^	2585 ± 51^a^	0.0263
Potassium (mg)	1988 ± 34^a^	2036 ± 39^a^	2095 ± 44^a^	2246 ± 44^b^	<0.0001
Calcium (mg)	597 ± 15^a^	637 ± 15^ab^	690 ± 17^b^	765 ± 19^c^	<0.0001
Iron (mg)	10 ± 0.2^a^	11 ± 0.2^a^	13 ± 0.2^b^	15 ± 0.3^c^	<0.0001
Magnesium (mg)	198 ± 4^a^	202 ± 4^a^	207 ± 5^a^	230 ± 5^b^	<0.0001
Zinc (mg)	7.6 ± 0.2^a^	7.6 ± 0.1^a^	8.1 ± 0.2^a^	9.3 ± 0.2^b^	<0.0001
Whole grain (servings/14 d)	6.1 ± 6^a^	7.3 ± 6^a^	8.8 ± 6^b^	12.3 ± 7^c^	<0.0001

^a,b,c,d^Means within the same row with the same letter are not significantly different (*P *< 0.01). *Adjusted for energy and age.

**Table 4:** Percentage of adults 55+ falling below the Estimated Average Requirement (EAR) by RTE cereal consumption pattern.

	Percent falling below EAR									
Nutrient	Men					Women				
	Q1	Q2	Q3	Q4	*P*	Q1	Q2	Q3	Q4	*P*
Vitamin A	58.4^a^	52.7^a^	35.8^b^	20.5^c^	<0.0001	55.2^w^	44.8^x^	34.9^y^	17.6^z^	<0.0001
Vitamin C	58.0^a^	53.1^a^	38.2^b^	40.0^b^	<0.0001	55.2^w^	50.2^wx^	44.2^xy^	35.6^y^	0.0002
Vitamin E	95.2^a^	96.1^a^	91.5^ab^	87.5^b^	0.0042	97.3^w^	97.8^w^	94.4^w^	95.6^w^	0.1833
Thiamin	14.7^a^	6.3^b^	1.8^bc^	0.5^c^	<0.0001	29.3^w^	15.2^x^	9.3^xy^	5.4^y^	<0.0001
Riboflavin	3.9^a^	1.0^b^	0.0^b^	0.0^b^	0.0018	13.1^w^	2.5^x^	0.5^x^	0.0^x^	<0.0001
Niacin	3.9^a^	3.9^a^	2.4^ab^	0.0^b^	0.1547	14.3^w^	8.7^x^	4.7^xy^	2.0^z^	<0.0001
Vitamin B6	40.7^a^	30.4^b^	11.5^c^	3.5^d^	<0.0001	68.0^w^	54.5^x^	32.6^y^	13.2^z^	<0.0001
Folate	26.4^a^	9.2^b^	1.8^c^	1.0^c^	<0.0001	54.1^w^	28.9^x^	11.6^y^	3.9^z^	<0.0001
Vitamin B12	6.9^a^	1.5^b^	0.6^b^	0.0^b^	<0.0001	19.7^w^	7.2^x^	3.7^xy^	1.0^y^	<0.0001
Iron	1.7^a^	1.5^a^	0.0^a^	0.0^a^	0.1161	3.1^w^	0.7^x^	0.0^x^	0.0^x^	0.0025
Magnesium	90.9^a^	89.9^a^	82.4^b^	69.5^c^	<0.0001	91.5^w^	82.0^x^	79.5^x^	67.8^y^	<0.0001
Zinc	52.8^a^	45.9^a^	32.1^b^	15.5^c^	<0.0001	50.6^w^	38.3^x^	25.1^y^	13.7^z^	<0.0001
Calcium	74.0^a^	67.2^a^	49.7^b^	34.0^c^	<0.0001	95.8^w^	89.5^x^	88.4^x^	77.1^y^	<0.0001
Vitamin D	95.7^a^	95.7^a^	95.2^ab^	87.0^b^	0.0010	99.2	98.9	98.6	99.0	0.9305

^abcd^Proportions within the same row with same letters are not significantly different (*P* < 0.01) (men).

^wxyz^Proportions within the same row with same letters are not significantly different (*P* < 0.01) (women).

**Table 5:** Adjusted* mean body mass index for adults 55+ years and percent overweight or obese by quartile of cereal consumption (BMI ≥ 25) by RTE cereal consumption pattern (energy excluded).

			Quartile of RTE cereal consumption			
		Q1	Q2	Q3	Q4	
		0 Servings	1–3 Servings	4–7 Servings	8+ Servings	
Gender/age	Sample size		Mean BMI			Overall *P*

Male ages 55+	803	28.19^a^	27.96^a^	27.36^ab^	26.93^b^	0.0332
Female ages 55+	956	27.48^ba^	28.22^ba^	28.53^a^	27.09^b^	0.0607

			Percent overweight/obese****			*P*

Male ages 55+	803	74.43^a^	70.69^a^	63.05^b^	64.35^b^	0.0433
Female ages 55+	956	61.11^a^	63.40^a^	62.57^a^	58.81^a^	0.7575

			Percent obese			

Male ages 55+	803	28.70^ab^	30.61^a^	24.42^ab^	21.02^b^	0.1111
Female ages 55+	956	28.63^a^	33.19^a^	32.46^a^	28.84^a^	0.5622

^a, b^ Values within the same row with the same letter are not significantly different (*P *< 0.05).

*Mean values adjusted for age, age^2^.
